# Switchable Pressure-Sensitive Adhesion in Nematic Side-Chain Liquid Crystal Elastomers

**DOI:** 10.1021/acs.macromol.5c01692

**Published:** 2025-10-09

**Authors:** Noboru Koshimizu, Mohand O. Saed

**Affiliations:** † Cavendish Laboratory, 2152 University of Cambridge, J.J. Thomson Avenue, Cambridge CB3 0HE, U.K.; ‡ Electronic and Imaging Materials Res. Laboratories, Toray Industries, Inc., 3-2-1 Sonoyama, Otsu 530-0842, Shiga, Japan

## Abstract

Switchable pressure-sensitive adhesives (PSAs) offer promising opportunities for reusability, on-demand debonding, and programmable adhesion. In this work, we report the design and synthesis of a novel thiol-functionalized liquid crystal mesogen, enabling the fabrication of side-chain nematic liquid crystal elastomer (SC-LCE) adhesives via siloxane-based thiol-ene click chemistry. These materials uniquely combine the thermomechanical responsiveness of LCEs with rheological profiles that approach the Dahlquist criterion for ideal PSAs. Specifically, the resulting SC-LCEs exhibit a glass transition temperature of 6 °C and a rubbery plateau modulus of 0.3 MPa, achieved without the use of plasticizers, tackifiers, or other additives. In particular, our SC-LCE adhesives exhibit exceptional viscoelastic energy dissipation, with a peak loss factor (tanδ) of 2.81, and maintain high damping (tanδ > 1) over a wide temperature range (0–45 °C) in the nematic phase. SC-LCE adhesives also exhibit tackiness comparable to that of high-performance commercial adhesives such as VHB tapes while maintaining full thermal switchability. Furthermore, the peel and lap shear forces are more than double those of conventional main-chain LCEs. These findings represent a significant advance toward the development of high-performance, thermally debondable, and fully reusable PSA systems with promising implications for sustainable and reconfigurable adhesive technologies.

## Graphical abstract

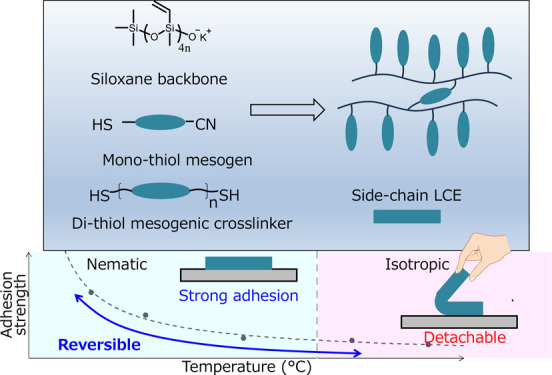

## 1 Introduction

Adhesives are ubiquitous in our daily lives, and are widely used in many vital everyday applications, such as seals, labels, protection and packaging sheets, biomedical, display, and electronic equipment.^1–3^ However, because of their single use and disposability, adhesives pose a series of challenges for waste management and environmental sustainability, with millions of tons produced annually.^4^ Most adhesives, especially pressure-sensitive adhesives (PSAs), are difficult or impractical to remove from substrates.^5,6^ This results in the removal of the adhesive that contaminates recycling parts and prevents even commonly recyclable plastics such as polyolefins, paper, and cardboard from being recycled. The current method for removing adhesive contaminants relies on yet another unsustainable chemical process, which involves swelling in harsh and expensive solvents (e.g., NaOH, amine-based solvents), followed by rigorous washing. Otherwise, adhesives are discarded in the environment or buried in landfills. This is because PSA materials primarily consist of nonrecyclable, cross-linked polymer networks.^7^

Reusable adhesives (chemically recyclable and cleanly detachable) offer a sustainable alternative to conventional single-use PSAs.^8,9^ Repurposing these adhesive materials reduces waste and the need for recycling, while providing greater flexibility in application design, particularly where debonding on demand is necessary. Such adhesives are also crucial in applications such as robotics, wearable electronics, and manufacturing assembly.^10,11^ These switchable adhesives can be triggered by environmental changes such as pH, solvent, temperature, mechanics, electro, and magnetic field.^12^ Currently, materials that exhibit reversible adhesion can be achieved via chemical functionality^13–15^ or topological microstructures^9,11,16–18^ When using chemical functionality, adhesion can be switched by modulating intermolecular force interactions, such as hydrogen bonding, dipole–dipole interactions, electrostatic forces, or hydrophobic interactions.^19,20^ This is typically achieved by using polymers that exhibit stimuli-responsive properties.^12,21–25^ On the contrary, the use of topological microstructures relies on patterning of the surface to maximize and minimize the contact area on demand.^9^ However, achieving reliable switchable adhesives still presents a major challenge, as adhesives with high adhesion strength are typically difficult to remove, while the current reusable adhesives often tend to be weaker.^16^ To overcome this challenge, the adhesive must be strong enough to match traditional PSAs and easy to remove.^18^ Generally, once the adhesive is bonded to a surface, the strength of the adhesive is determined by its ability to resist crack propagation through internal energy dissipation.^26^ However, to achieve a strong bonding, good contact must be established between the adhesive and the substrate. This is largely governed by the thermomechanical properties of the material as outlined in the Dahlquist criterion.^27–29^ Essentially, the Dahlquist criterion requires the adhesive to be permanently tacky at the working temperature, ideally with a glass transition temperature (*T*_g_) below 0 °C and a shear storage modulus (*G′*) of less than 0.1 MPa at a frequency of 1 Hz. Together, high and controllable dissipation and tackiness (i.e., wettability) are needed to achieve optimum switchable adhesives.

Recently, we utilized the intrinsic phase change behavior in liquid crystalline elastomers (LCEs) to enable fast, switchable adhesion.^30–34^ The concept of switchable adhesion in nematic LCEs was first theoretically proposed by Adams et al. in 2012, where they found that the tack force in the nematic phase more than doubled when transitioning to the isotropic phase.^35^ The enhanced switchable adhesion phenomenon LCEs is attributed to the inherent high-energy dissipation in the nematic region, where adhesion is strong, and low dissipation in the isotropic region, where adhesion is weak.^30^ The high dissipation observed in nematic LCE materials results from the internal rearrangements of liquid crystalline molecules (i.e., mesogens) within the polymer backbone under small but nonzero stress.^36^ Despite the great potential and clear advantages of LCE switchable adhesives, these materials have not been optimized to match the thermomechanical behavior of traditional ’ideal’ PSA materials in two key aspects: (a) Matching the Dahlquist criterion for ideal PSAs: This requires achieving a material that is permanently and aggressively tacky at room temperature, as mentioned earlier. Specifically, *G′* must be below 0.1 MPa at room temperature to ensure sufficient surface conformity and tack.^35,37^ However, current LCE adhesives, primarily main-chain (MC) LCEs, struggle to meet this criterion. Even when *T*_g_ is tuned to around 0 °C, the presence of stiff mesogenic units in the polymer backbone results in a *G′* typically between 5 and 10 MPa at room temperature.^30,34,38,39^ This is 2 orders of magnitude higher than the Dahlquist threshold, severely limiting tack performance. Previously, MC-LCEs required annealing to temporarily reduce G′ and achieve high tack.^30,32–34^ (b) Maximizing internal viscoelastic dissipation: Ideal PSA materials should exhibit a high tan δ, typically in the range of 0.5–1.0 at application-relevant frequencies (1–100 Hz), which enables efficient energy dissipation during debonding and improves adhesive performance. However, MC-LCEs often display significantly lower tan δ values compared to SC-LCEs due to their relatively limited molecular mobility. This gap in viscoelastic dissipation reduces their ability to absorb and dissipate mechanical energy, which is critical for applications requiring robust adhesion and controlled debonding.^40^

In this study, we developed new SC-LCE switchable PSAs. The SC-LCE adhesive materials were optimized to meet the Dahlquist criterion while maximizing internal viscoelastic dissipation to enhance adhesive strength. SC-LCEs are more flexible and exhibit higher dissipation compared to MC-LCEs because the mesogens attached as side groups to the polymer network provide greater flexibility and freedom of movement, allowing for enhanced viscoelastic energy dissipation.^40–42^ However, traditional SC-LCEs are notoriously difficult to synthesize because they typically rely on hydrosilylation reactions.^43^ The hydrosilylation reaction is based on a noble platinum catalyst, which is highly sensitive to moisture and can be easily poisoned by even minor changes in the reaction environment. Additionally, Si–H bonds are susceptible to oxidation, hydrolysis, and unintended polymerization, making it difficult to reliably scale up these reactions.^43–45^ Building on the recent success of thiol–ene click chemistry in developing MC-LCEs with highly controlled network architectures, it has significantly improved their accessibility.^46–48^ We employed the thiol–ene ‘click’ reaction to synthesize SC-LCE nematic materials with tunable thermomechanical properties and enhanced processability. The thiol–ene reaction has previously been employed to successfully synthesize LCE materials, including MC-LCEs and SC-LCEs, for actuation purposes^46–49^ However, the resulting SC-LCEs exhibited a smectic liquid crystalline (LC) phase and were never optimized for adhesion applications.^50^ Here, we employed vinyl-functionalized siloxane polymer backbones, where the siloxane linkages in the polymer backbone enable softness, flexibility, and the ability to recycle the polymer networks via siloxane equilibration.^48,51^ The vinyl-functionalized siloxane was then polymerized with newly designed monofunctional and difunctional thiol-based mesogens and cross-linkers to finely tune the rheological properties of the SC-LCE adhesive. This approach allowed us to fabricate LCE materials that closely satisfy the Dahlquist criterion for ideal PSAs. Thus, our SC-LCE adhesive exhibited a *T*_g_ of 6 °C and a rubbery storage modulus (*E*′) of 0.3 MPa, comparable to that of commercial PSAs such as VHB tapes.

## 2 Experimental Section

### 2.1 Materials

4-Cyanophenol was purchased from Thermo Scientific. 4-(bromomethyl)­benzoic acid from Tokyo Chemical Industry Co., LTD (TCI). 2,4,6,8-Tetramethyl-2,4,6,8-tetravinylcyclotetrasiloxane, *N*,*N*′-Diisopropylcarbodiimide (DIC), 4-Dimethylaminopyridine (DMAP), 1 M hydrochloric, 2,2′-(Ethylenedioxy)­diethanethiol (EDDET), 2,6-Di*tert*-butyl-4-methylphenol (BHT), dipropylamine (DPA) and 2,2-Dimethoxy-2-phenylacetophenone (I-651) were purchased from Sigma-Aldrich. Thiourea was purchased from Merck. Potassium metabisulfite was purchased from East Anglia Chemicals Limited. [4-(3-Acryloyloxypropyloxy)­benzoic acid 2-methyl-1,4-phenylene ester diacrylate], RM257 was obtained from Daken Chemical Ltd. Tetrahydrofuran (THF), dichloromethane, and toluene were purchased from Sigma-Aldrich and used as solvent.

### 2.2 Preparation of Monothiol Mesogen

4-Cyanophenyl 4-(mercaptomethyl)­benzoate was synthesized by the following steps as shown in Scheme 1a. 4-Cyanophenyl 4-(bromomethyl)­benzoate (was prepared by esterification of 4-cyanophenol with 4-(bromomethyl)­benzoic acid. In summary, reaction of 4-cyanophenyl 4-(bromomethyl)­benzoate with thiourea affords the isothiuronium salt which upon basic hydrolysis generates 4-cyanophenyl 4-(mercaptomethyl)­benzoate. 4-cyanophenol (14.01 g, 117.65 mmol) and 4-(bromomethyl)­benzoic acid (25.30 g, 117.65 mmol) were dissolved in THF (200 mL) in a 500 mL single necked flask. The mixture was cooled to 0 °C followed by the addition of DIC (16.33 g, 129.41 mmol) and DMAP (1.61 g, 13.18 mmol) and stirred for 30 min. The mixture was warmed to room temperature and stirred overnight. The white solid was filtered from the mixture, washed with 1 M hydrochloric acid (150 mL), filtered and dried to give the white powder of 4-cyanophenyl 4-methylbromobenzoate (25.77 g, 69.3%). 4-cyanophenyl 4-(methylbromo)­benzoate (8.60 g, 27.20 mmol) and thiourea (10.35 g, 136.01 mmol) were dissolved in THF (250 mL) in a 500 mL single-neck flask and refluxed for 48 h. THF was removed under reduced pressure. The white solid was filtered from the mixture, washed with acetone and dried followed by the addition of thiourea (4.14 g, 54.40 mmol), potassium metabisulfite (60.47 g, 272.01 mmol), water (200 mL) and dichloromethane (200 mL) in a 1000 mL single-neck flask. The mixture was refluxed for 12 h, and dichloromethane was removed under reduced pressure. The white solid was filtered from the mixture, recrystallized from hexane, and dried to give a white powder of 4-cyanophenyl 4-(mercaptomethyl) benzoate (2.44 g, 33.39%).

**Scheme 1:**
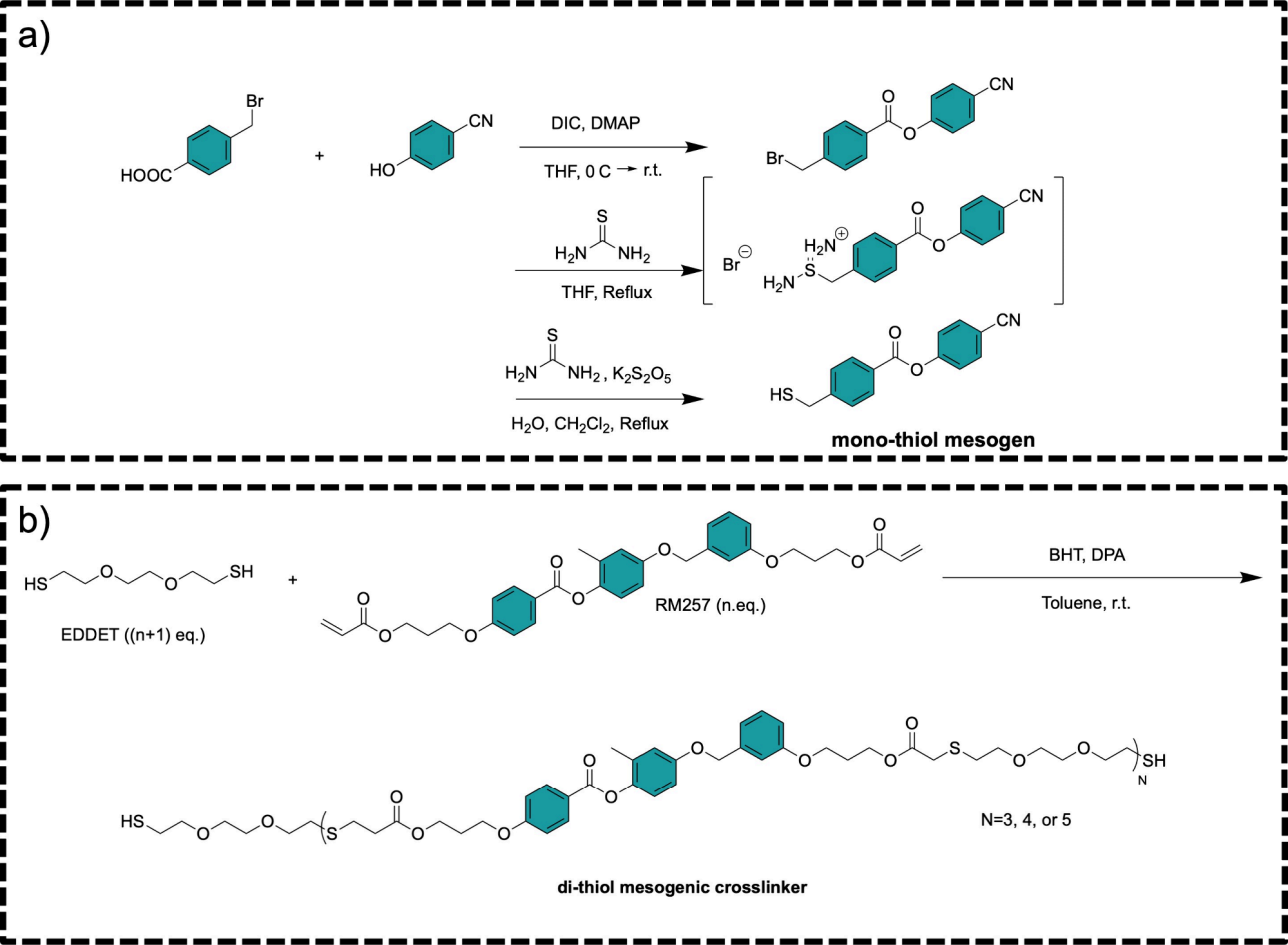
(a) Synthesis of 4-Cyanophenyl 4-(Mercaptomethyl)­benzoate (Monothiol Mesogen); (b) Synthesis of Dithiol Mesogenic Cross-Linker (Cn)

### 2.3 Preparation of the Cross-Linker

The controlled chain length cross-linker, terminated with thiol groups at the ends, was synthesized through the following steps as shown in Scheme 1b. RM257 was dissolved in toluene (60% by weight relative to RM257) with an excess amount of EDDET and BHT (2% by weight relative to RM257) in a vial assisted by gentle heating and vigorous mixing. DPA (0.5 wt % relative to RM257) was then added and thoroughly mixed. The reaction mixture was allowed to react for 24 h at room temperature. Thiol-terminated oligomers designated as Cn, where n is the number of mesogenic monomers, were prepared in this way by adjusting the molar ratio of RM257 and EDDET.

### 2.4 Preparation of Side-Chain LCE (SCn)

*SCn* was synthesized through the following steps as shown in Figure 1. 2,4,6,8-Tetramethyl-2,4,6,8-tetravinylcyclotetrasiloxane and 1.28 wt % potassium trimethyl silanolate were mixed and heated in a vial at 85 °C for 1 day to obtain liquid of siloxane prepolymer Scheme S1. The siloxane prepolymer (0.10 g), 4-cyanophenyl 4-(mercaptomethyl) benzoate (3.6 equiv) and Cn (0.2 equiv) were added toluene (2.20 g) in a vial assisted by gentle heating and vigorous mixing. The mixture was cooled to room temperature, added I-651 (0.5 wt % relative to all components except toluene) and irradiated with UV (365 nm wavelength) for 3 h. Then, toluene was removed under reduced pressure. The adhesive tapes were fabricated from *SCn* samples. This was done postpolymerization using a hot-pressing machine (Moore hydraulic press). Specifically, *SCn* samples were sandwiched with a paper spacer of 0.2 mm thick paper spacer between a 23 μm PET film and silicone release paper to create a 0.2 mm thick adhesive tape. Alternatively, two sheets of silicone paper were used to prepare a free-standing adhesive film of the same thickness. The hot pressing was performed at 100 °C and 10 MPa for 2 h. Poly­(methylvinylsiloxane) contains an oxygen anion at the chain end, which facilitates siloxane equilibration during hot pressing, enabling reprocessing.^51^

**Figure 1:**
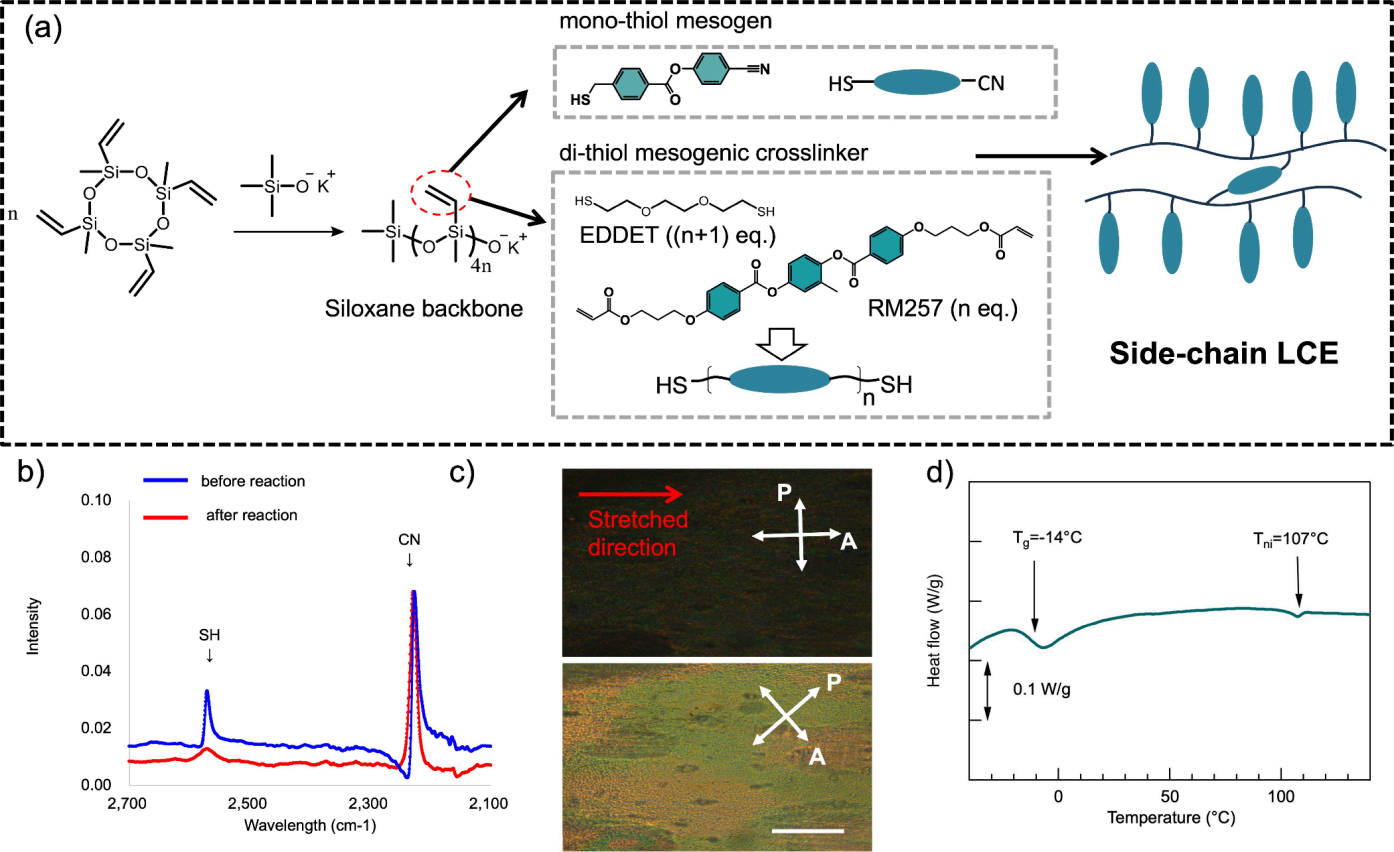
(a) Synthesis of *SCn*. The siloxane ring of 2,4,6,8-tetramethyl-2,4,6,8-tetravinylcyclotetrasiloxane is opened by addition of anionic catalyst and heating to create poly­(methylvinylsiloxane) oligomer having an oxygen anion at the end. 90 and 10 mol % of vinyl groups of poly­(methylvinylsiloxane) reacted with monofunctional thiol mesogen and difunctional thiol oligomer “crosslinker.” (b) FTIR spectra of monofunctional thiol mesogen in *SC3* normalized at the cyano peak. The progress of thiol–ene photo reaction was confirmed by the peak decrease of thiol group. (c) POM observations of the 100% stretched *SC3* free-standing film. It shows darkness when the stretch direction and analyzer direction are parallel. It turns to brightness when the stretched director is rotated 45 deg from the analyzer direction. (d) DSC curve of a *SC3* free-standing adhesive films.

### 2.5 Preparation of MC, Adhesive Tape, and Free-Standing Film

MC was synthesized through the following steps as shown in Scheme S2. The synthesis and preparation, including the adhesive tape, were carried out following our previously reported method.^32^

### 2.6 Dynamic Scanning Calorimetry (DSC)

DSC, (DSC4000 PerkinElmer), samples with approximately 10 mg were loaded into standard aluminum DSC pans. The samples were heated to 150 °C at 10 °C min^–1^, held isothermally for 5 min, cooled to −50 °C at 10 °C min^–1^, and held isothermally for 5 min. This cycle was repeated three times, and a third heating cycle was used to define *T*_g_ and *T*_ni_.

### 2.7 Dynamic Mechanical Analysis (DMA)

Dynamic mechanical tests were performed on a Discovery DMA850, TA Instruments, in tension modes, with free-standing samples of thickness 0.2 mm for *SCn* and MC and 1.0 mm for VHB. A fixed frequency of ω = 1.0 Hz and a strain amplitude of 0.02% were applied and data were acquired on heating at the rate of 3 °C min^–1^ from −50 to 150 °C.

### 2.8 Probe Tack Test

A 10 mm diameter spherical stainless steel probe was mounted on a Tinius Olsen vertical dynamometer frame. The probe was cleaned with acetone and lowered to the adhesive surface on a rigid glass substrate that was glued onto a flat rigid base on a hot plate that controls the sample temperature. The adhesive film (thickness 0.2 mm for *SC3* and *MC* and 1.0 mm for VHB) supported by a glass slide was prepared by placing silicone paper on the film and applying pressure at 110 °C for 20 min. The tests were carried out under controlled *T* by the hot plate. Once a fixed load of −0.5 N was reached, a predetermined dwell time (300 s) was allowed while maintaining the same compression. The probe was then pulled up at a fixed rate of 10 mm min^–1^ while recording the force generated. Each test was repeated at least three times and the average of the maximum force reads for detachment was determined as *F*_ad_ for each condition.

### 2.9 180 Degree Peel Test

Peel tests were performed using a 180 ° peel geometry on a Discovery DMA850 (TA Instruments). Due to spatial constraints within the testing chamber and limitations in speed and force, LC adhesive tapes with a width of 5 mm were used, and the pulling rate was set at 100 mm min ^–1^. A 5 mm wide adhesive tape, prepared with a PET film as described in Section 2.5, was applied to a stainless steel substrate under gentle pressure at 110 °C for 20 min, forming a three-layer structure comprising the stainless steel plate, the adhesive layer, and the PET film. The tape was then allowed to cool to room temperature. The free end of the tape was folded back and clamped into the upper vertical frame of the dynamometer, while the substrate plate was secured in the lower clamp. The temperature was held isothermally for 5 min prior to testing. The tape was then peeled upward at a constant rate of 100 mm min-1 and the adhesion force was recorded. The measured force was normalized by the width of the tape. Each test was repeated at least three times and the average of the maximum normalized force was reported.

### 2.10 Lap Shear Test

The free-standing adhesive films were cut to a fixed area (10 × 10 mm^2^) and sandwiched between two stainless steel plates. The lap shear test was conducted according to ASTM D5656, and the shear strain (%) is calculated using the formula:

$$\mathrm{Shear}\;\mathrm{strain}\;(\%)=x=\frac{\mathrm{Displacement}\;(\mathrm{mm})}{\mathrm{Thickness}\;(\mathrm{mm})}\times 100$$

The sample was applied pressure at 110 °C for 20 min. Both slides were clamped to the vertical dynamometer frame on the Tinius Olsen ST1 tensiometer and a shear deformation is imposed on the adhesive layer at a speed of 1.25 mm min^–1^. The curve at high temperature was obtained by heating a heat gun during the test.

### 2.11 Lap Shear Demonstration

To better visualize the adhesion property on demand, especially its maximum shear stress, a demonstration was made using a 10 × 10 mm *SC3* or VHB samples that were sandwiched between two stainless steel plates with 10 mm overlap and annealed as described in Section Lap Shear. The upper steel plate was fixed to a frame and the lower steel plate was hung with a weight of 1 kg. Once the weight was lifted, the steel plates (and thus the adhesive film) were heated.

## 3 Results and Discussion

The purpose of this study is to develop switchable LCE-based adhesives that satisfy the Dahlquist criterion while maximizing internal viscoelastic dissipation. A siloxane backbone is advantageous for SC-LCEs, as it lowers the *T*_g_ as a result of the flexible Si–O linkages. However, the high incompatibility with carbon-based mesogens often leads to the formation of smectic phases.^50^ Nematic LCs align only along the long axis of the mesogen (director), whereas smectic LCs exhibit a layered structure in which the mesogens align perpendicular to the layer plane, with the backbone and spacers compacted into thin sheets between the layers. This smectic structure restricts the mobility of mesogens, resulting in reduced dissipation compared to that of nematic LCs. Therefore, achieving both a low *T*_g_ and a nematic phase simultaneously in conventional SC-LCEs has been challenging. In this study, a short spacer and a polarized cyano group were introduced at the end of the side chain to disrupt the formation of the layered smectic structure. The synthesis of 4-cyanophenyl 4-(mercaptomethyl)­benzoate (monofunctional thiol mesogen) is presented in Scheme 1. Figure 1 illustrates the synthesis route and characterization of SC-LCEs. To obtain SC-LCEs, we employed a thiol–ene click reaction, as opposed to the traditional hydrosilylation reaction commonly used for SC-LCE synthesis. The hydrosilylation reaction requires a noble platinum catalyst and is sensitive to moisture, whereas the thiol–ene click reaction proceeds under mild conditions using a cost-effective starting material, making it more suitable for scale up and industrial applications.^43,52^ First, poly­(methylvinylsiloxane) was synthesized by adding 1.28 wt % potassium trimethyl silanolate to 2,4,6,8-tetramethyl-2,4,6,8-tetravinylcyclotetrasiloxane and heating at 85 °C overnight (Scheme S1, Supporting Information). Poly­(methylvinylsiloxane) was then polymerized with monofunctional and difunctional thiol-based mesogens and cross-linkers through thiol–ene photopolymerization (Figure 1a). The difunctional thiol mesogenic cross-linker was synthesized via a self-limiting thiol–acrylate Michael addition between a nematic diacrylate and an isotropic dithiol. Next, 0.9 equiv of monofunctional thiol mesogen and 0.1 equiv of cross-linker were reacted with 1.0 equiv of the vinyl group of poly­(methylvinylsiloxane) via the thiol–ene photopolymerization reaction, yielding SC-LCEs designated as *SCn*, where n represents the proportion of mesogenic monomers in the cross-linker. The progress of the reaction was confirmed by FTIR that the peak decrease of the thiol group (Figure 1b). LC phase and phase transition temperatures were characterized using a polarized optical microscope (POM) and differential scanning calorimetry (DSC) (Figure 1c,d). The nematic phase was identified by POM images; we used an aligned free-standing film of *SC3*. The sample shows darkness when the stretching direction and the analyzer direction are parallel. Brightness appears when the stretched director is rotated 45 deg from the analyzer direction, confirming the optical anisotropy characteristic of the nematic LCE sample. The isotropic phase was also confirmed by the disappearance of brightness in the POM observation upon heating above 110 °C. Thermomechanical properties were optimized by adjusting the amount of potassium trimethylsilanolate to achieve high viscoelastic dissipation (Table S1, Supporting Information). An example of the DSC curve for an *SC3* sample shows a *T*_g_ around −14 °C and a nematic-to-isotropic transition temperature (*T*_ni_ *)* around 107 °C. The combination of low *T*_g_ and high *T*_ni_ enables for a wide range of the nematic phase, allowing for a broad range of adhesive working temperatures. The DSC curves for other SC-LCE formulations can be found in Figure S5 and Table S1 in the Supporting Information.

Before testing, adhesive tapes were manufactured from *SCn* samples (Scheme 2). This was done postpolymerization using a hot-pressing machine (Moore hydraulic press). Specifically, *SCn* samples were sandwiched with a 0.2 mm thick paper spacer between a 23 μm polyethylene terephthalate (PET) film and silicone release paper to create a 0.2 mm thick adhesive tape. Alternatively, two sheets of silicone paper were used to prepare a free-standing adhesive film of the same thickness. The hot pressing was performed at 100 °C and 10 MPa for 2 h. Poly­(methylvinylsiloxane) contains an oxygen anion at the chain end, which facilitates siloxane equilibration during hot pressing, enabling reprocessing (schem2a). It should be noted that although the SCn samples are elastomers and can recover from creep at room temperature (see Figure S10 in the Supporting Information), they can still be reshaped under mild conditions (100 °C under 20-ton pressure for 2 h) due to siloxane equilibration via covalent bond exchange during hot pressing.^51^ This condition is significantly milder compared to the high temperatures required to reshape traditional siloxane elastomer networks such as PDMS or siloxane LCEs, which typically require temperatures above 200 °C.^48,53^ This reshaping capability is not present in traditional PSAs, which lack the intrinsic dynamic covalent chemistry of our LCE system.

**Scheme 2:**
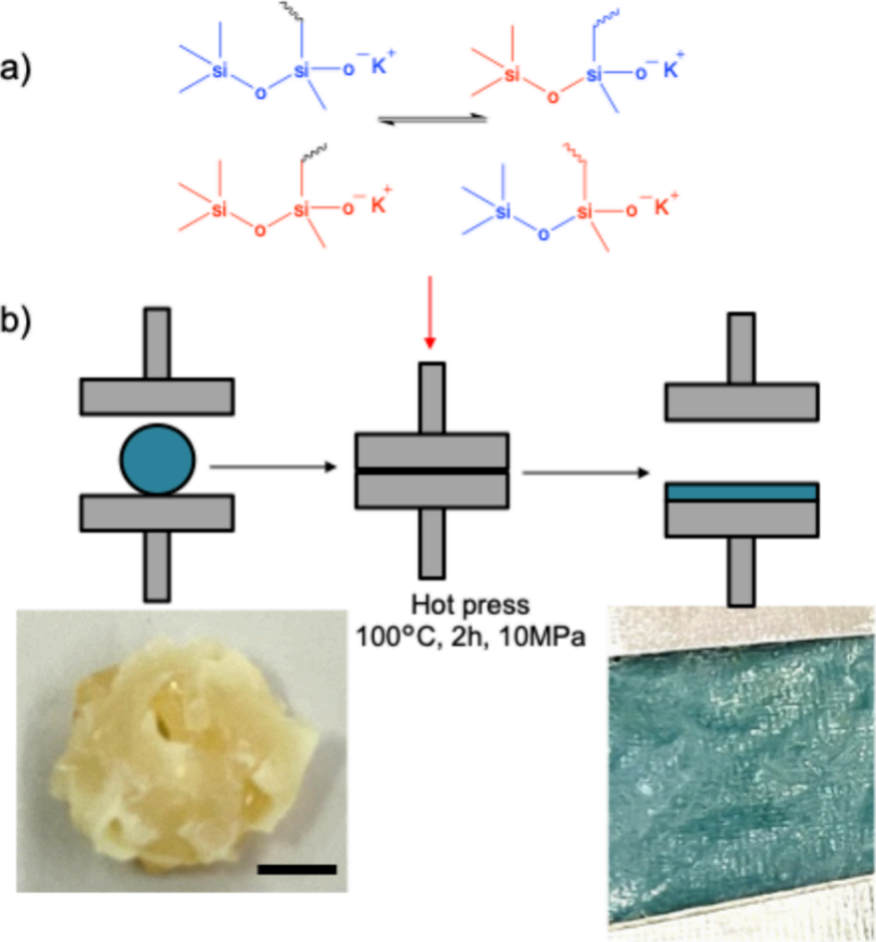
(a) Siloxane Equilibration Mechanism via Anionic Catalysis during Hot Pressing; (b) Image of Cut and Reassembled *SC3* Sample Prior to Hot Pressing, and the Reshaped 0.2 mm Thick *SC3* Film after Hot Pressing^a^

a The scale bar is 10 mm.

Controlling the thermomechanical properties of the adhesive, such as *T*_g_ and *E′*, is critical and can be achieved by varying the concentration of mesogen in the cross-linker. The thermomechanical properties of our *SCn* samples, along with control samples from traditional MC-LCEs and a commercially available free-standing adhesive film (VHB tape from 3M), were measured using Dynamic Mechanical Analysis (DMA) (Figure 2a,b). As expected, SCn samples exhibit lower *E′* and a higher loss factor (tan δ) compared to MC-LCE and VHB tape, while maintaining a similar *T*_g_ (5–10 °C). This behavior is attributed to the increased mobility of mesogens in SC-LCEs compared to that of MC-LCEs. It is important to note that DMA tests generally produce higher *T*_g_ values compared to DSC measurements (−15 to −5 °C higher). This discrepancy arises from the inherent differences between these techniques, including the positioning of the temperature sensor, insulation, heating rates, and sample sizes. *E′* decrease*s* and tan δ increases with decreasing mesogen content in the cross-linker. This trend is attributed to the higher proportion of monofunctional thiol (side-chain) mesogens relative to difunctional thiol (main-chain) mesogens in the polymer network. The respective proportions of monofunctional and difunctional mesogens are 53.1 and 27.3 wt % for *SC3*, 48.8 and 33.0 wt % for *SC4,* and 45.3 and 37.8 wt % for *SC5*. These results are consistent with those obtained from classical nematic side-chain LCEs cross-linked with main-chain LCEs.^54,55^ The *E′* of *SC3* is 0.27 MPa at 20 °C, satisfying the Dahlquist criterion (*E′* = 0.3 MPa, equivalent to *G′* = 0.1 MPa). In particular, the *E’* of *SC3* is at least 1 order of magnitude lower than that of both MC and VHB, highlighting its superior flexibility and compliance. It is important to note that SCn DMA curves could not be obtained at high temperatures because *E′* decreased significantly as the temperature approached the nematic-to-isotropic transition temperature (for example, *SC3* showed *E′* of just 0.04 MPa at 90 °C). As the temperature increased further, the *E′* fell below the detection limit of the DMA instrument. The sharp decrease near the transition temperature is characteristic of dynamic soft elasticity.^36,40,56,57^ Tan δ, a measure of damping and energy dissipation, is directly related to adhesive performance.^37^ In adhesive systems, particularly PSAs, a moderate to high tan δ enhances tack and peel strength by facilitating energy dissipation during contact and separation. However, the optimal tan δ value depends on the application. Although higher values of tan δ values can improve damping performance, they can also increase the likelihood of adhesive failure, which is especially relevant when clean debonding without residue is desired. *SC3* exhibits a maximum tan δ of 2.81 across a broad temperature range, significantly higher than both MC and VHB. To our knowledge, such high tan δ values have not been previously reported for LCE-based PSAs. In particular, *SC3* satisfies the Dahlquist criterion without any additives, whereas commercial PSA tapes such as VHB typically require multiple additives to fine-tune properties such as *E′*, *T*_g_, and tan δ to optimize adhesion performance. Commercial PSAs typically contain additives such as tackifiers, plasticizers, and stabilizers (eg, antioxidants). These additives play a crucial role in tuning the viscoelastic properties and enhancing the weatherability. Tackifiers and plasticizers are commonly used to adjust *E′* and *T*_g_, while antioxidants serve as stabilizers to improve thermal and oxidative stability.^58,59^

**Figure 2:**
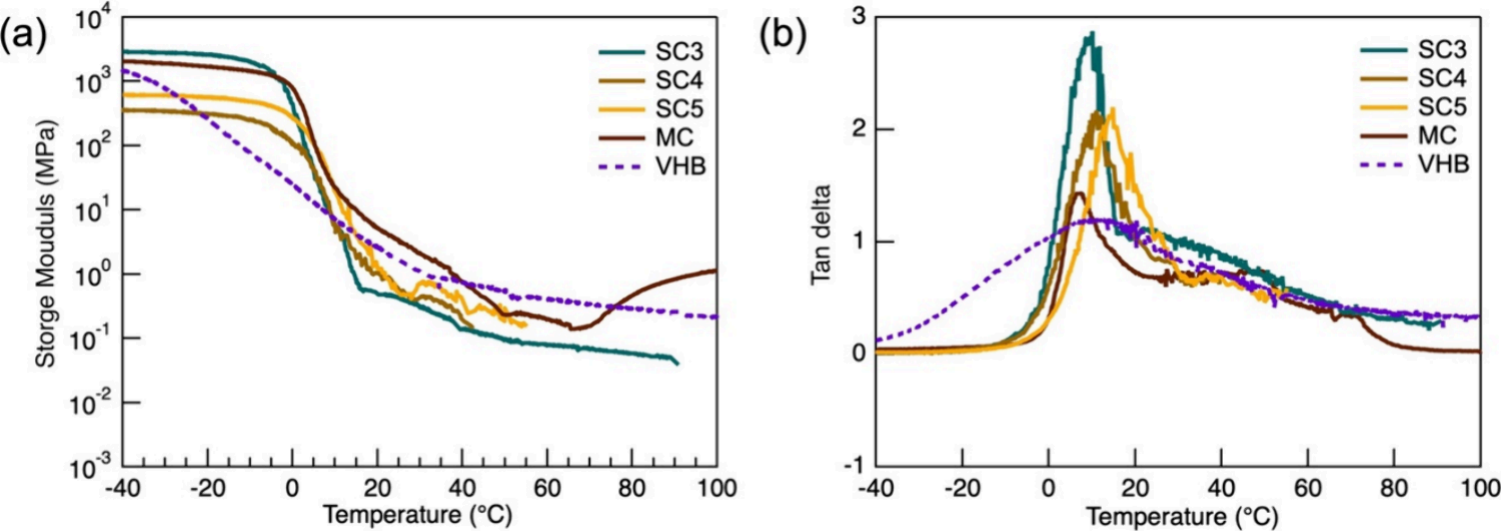
Comparison of the viscoelastic and adhesive properties of *SCn*, MC-LCE, and VHB tape. (a) Storage modulus (*E′*) and (b) loss factor (tan δ) were measured at a fixed frequency of ω = 1.0 Hz and a strain amplitude of 0.02%, during heating at a rate of 2 °C min.

A key feature of switchable adhesives is their ability to be activated (i.e., detached) on demand. They should exhibit low adhesion when detachment is required, while maintaining strong adhesion when bonded to the substrate. LCE adhesives change heating, transitioning from the nematic phase with high tan δ (high-energy dissipation) to the isotropic phase with low tan δ (low energy dissipation). Adhesive performance is typically evaluated using tack, shear, and peel tests.^60^ These properties correspond to different frequencies and types of external forces encountered in practical applications. Therefore, it is essential for a practical adhesive material to exhibit high adhesion strength across all three metrics, not just in one. To assess the temperature dependence of adhesion tackiness, a probe tack test was first performed, as this method is specifically designed to measure the adhesion adhesion tackiness (Figure 3a). At room temperature, *SC3* exhibited an adhesion force of 3.65 N. This is 1.5 times larger than that of MC and comparable to VHB, although the contact area of SC3 is 1.7 times smaller than that of VHB due to its reduced thickness (0.2 mm for *SC3* vs 1.0 mm for VHB). VHB is a commercially available 3 M tape with a fixed thickness of 1 mm. We fabricate our LCE tapes using a desktop blade coater or hot pressing, which limits the maximum achievable thickness to approximately 300 μm. Due to this constraint, we cannot produce LCE tapes with a thickness comparable to that of VHB. However, the probe tack tests were performed using a constant preload force, and the contact area between the probe and the sample was monitored via video imaging. When the same preload is applied, thinner films behave as stiffer materials due to a lower compliance ratio (*C*/*C*0), resulting in reduced deformation under load. This leads to a smaller contact depth and area compared to thicker films.^61–63^ In our case, *SC3*, despite being thinner, showed higher adhesion than MC and a performance comparable to VHB. The smaller contact area observed for *SC3* is consistent with the reduced compliance expected from its lower thickness. Although the probe size was constant, the actual contact area depends on the film’s ability to deform under load, which is influenced by its thickness and mechanical properties. Above *T*_ni_ at 110 °C, the adhesion force of *SC3* and MC decreased to 0.77 and 1 N. In contrast, VHB maintained a relatively high adhesion force of 1.26 N at 109 °C (Figure 3b). This pronounced reduction is mainly attributed to the phase transition in *SC3* from the nematic to the isotropic state, in addition to viscoelastic softening. Unlike VHB, which undergoes only viscoelastic changes with temperature, *the nature of* liquid crystal in SC3 introduces a unique thermal response. The phase transition disrupts molecular alignment and network integrity, leading to rapid loss of adhesive strength.^30^ It should be noted that all probe tack measurements were performed without annealing, whereas MC-LCEs typically require annealing to overcome poor tackiness.^30,32,34^ Moreover, to the best of our knowledge, this is the first demonstration of switchable adhesives that exhibit tackiness comparable to that of high-performance commercial adhesives such as VHB tape.

**Figure 3:**
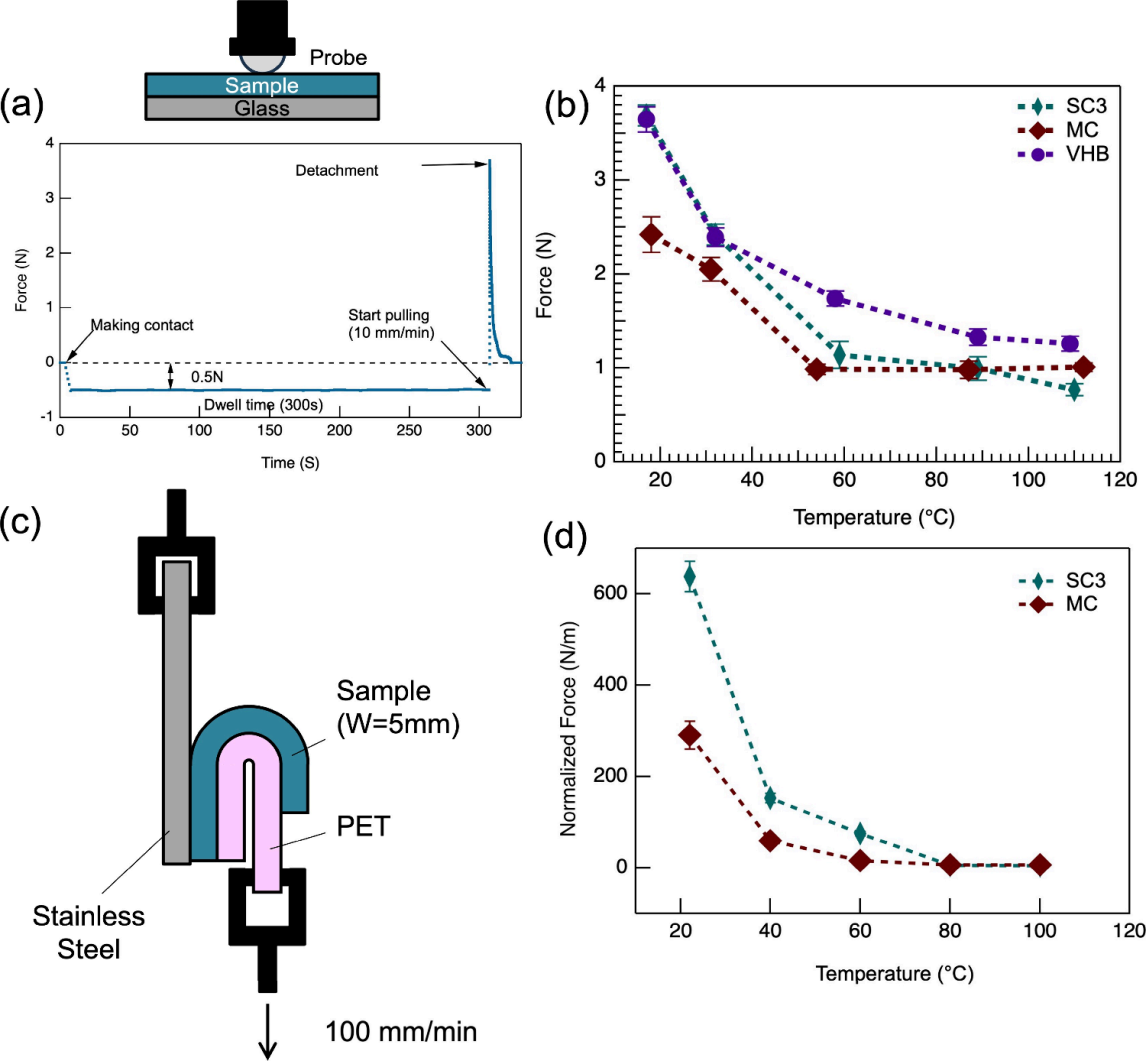
(a) Schematic of the probe tack test is shown. In this test, a stainless steel sphere probe (10 mm diameter) is brought into contact with the surface of the sample, mounted on glass under a load of 0.5 N for 300 s. The probe is then retracted at a rate of 10 mm min^–1^ while monitoring the force. A positive force indicates the amount required to detach the probe from the sample surface. (b) Plot shows force (Fa) as a function of temperature. (c) Schematic illustration of the 180° peel test setup. The force is recorded as the PET film is peeled at a rate of 100 mm/min. Normalized peel forces are defined as the maximum values obtained from the force–displacement curves. (d) Plot of normalized peel force versus temperature. Each test was repeated three times (*n* = 3).

The peel test is typically used to measure the force needed to break the bond between an adhesive and the substrate.^64^ A 180° peel test was performed to assess the temperature dependence of the normalized force (Figure 3c). For comparison, MC-LCE adhesive tapes and free-standing films were fabricated using a tape forming coater (MSK-AFA-III) from MTI Corporation (see Section 2).^32^At room temperature, *SC3* demonstrates a high normalized force of 638 N/m (Figure 3b), which is 2.5 times higher than MC. On the contrary, the normalized force of *SC3* is almost zero at high temperature (110 °C). We could not compare the peeling force of our *SCn* adhesives with that of commercial VHB tape due to differences in their thickness. However, for reference, we have included the peel force data for both VHB and LCE tapes in Figure S7 of the Supporting Information, despite the difference in their thicknesses.

The lap shear test is a standard method for evaluating the adhesive’s ability to resist lateral forces resulting from substrate sliding.^2^ High adhesion combined with easy-release functionality is critical for many applications. Here, we use the lab shear test to assess the temperature dependence of the adhesion strength in our samples (Figure 4a). *SC3* adhesive exhibits a high shear strength of 0.17 MPa at room temperature (Figure 4b), which is approximately 2.5 times higher than the MC-LCE adhesive. However, the SC3 shear strength drops to nearly zero at 110 °C in the isotropic phase. This is attributed to the significant decrease in tan δ. In particular, the lap shear test was performed without any annealing; the samples were simply placed in contact with the substrate and subjected to a slight pressure before testing. To demonstrate the switchable adhesive performance of *SC3*, a small piece of the adhesive film (10 × 10 cm) was sandwiched between two stainless steel plates and annealed at 110 °C for 5 min. The sandwiched setup lifted a weight of 1 kg easily at room temperature, and the adhesive film could be debonded from the stainless plate quickly once it was heated by a heat gun and transited to the isotropic phase. In comparison, the setup using VHB instead of *SC3* could not be debonded even if it was heated enough by a heat gun (see Figure S8 and the attached video in the Supporting Information). Developing switchable adhesives with lap shear strength comparable to high-performance commercial adhesives, such as VHB tape, represents an important step toward creating fully reusable and debondable adhesives without compromising performance.

**Figure 4:**
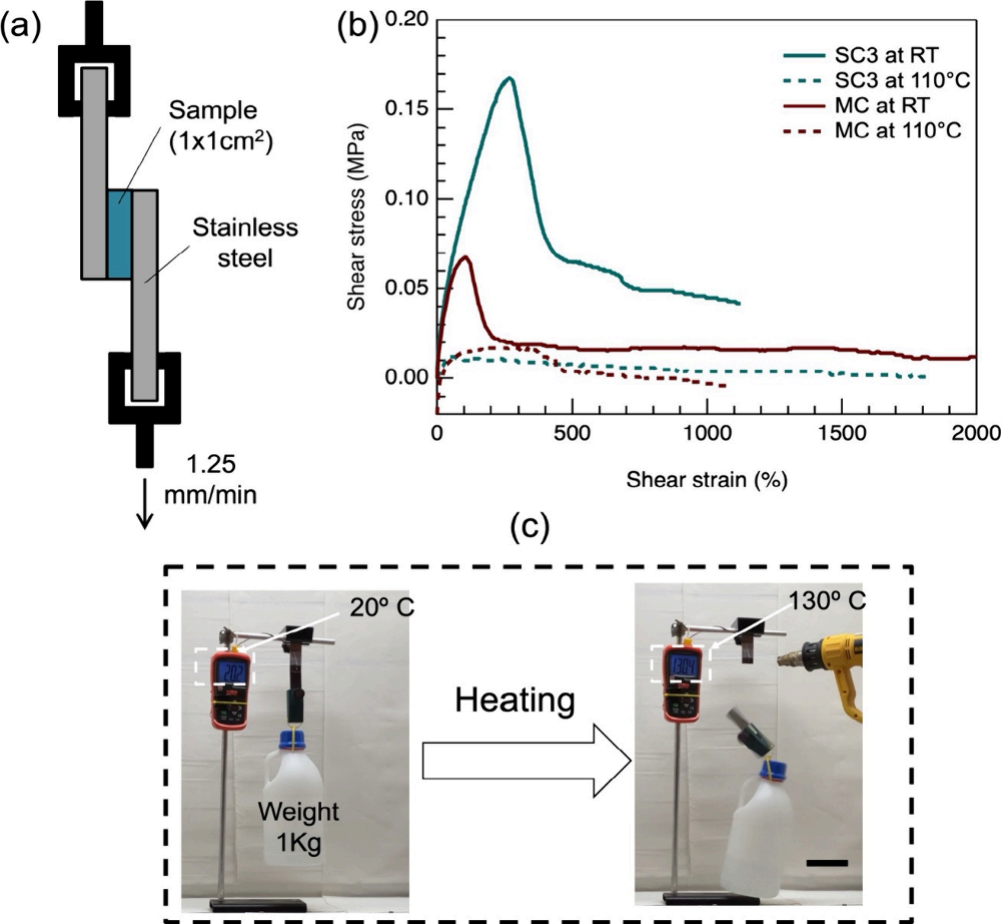
Lap shear testing as a function of temperature. (a) Schematic illustration of the lap shear test setup using a 10 mm wide adhesive film sandwiched between two stainless steel plates with a 10 mm overlap. One plate is fixed to a clamp while the other is pulled apart at a rate of 1.25 mm min^–1^. (b) Shear stress vs shear strain curves recorded at room temperature in the nematic phase and at 110 °C in the isotropic phase. (c) Demonstration of a double-sided, free-standing SC3 adhesive film (0.2 mm × 10 mm × 10 mm) lifting a 1 kg weight and detaching upon heating to the isotropic phase using a heat gun. The scale bar is 75 mm.

## 4 Conclusion

In this study, we developed the first SC-LCE adhesive that closely satisfies the Dahlquist criterion for ideal PSAs. The adhesive material was synthesized via click chemistry between thiol–ene vinyl groups on a siloxane backbone, monothiol mesogens, and mesogenic cross-linkers. The incorporation of an anionic catalyst enabled reprocessability through siloxane bond equilibration. SC-LCE adhesive exhibits high damping (tan δ = 2.81 at *T*_g_) and strong adhesion (3.65 N in the probe tack test) in the nematic phase, which is comparable to VHB, while showing three times lower adhesion (0.77 N) in the isotropic phase. Compared to MC-LCE adhesive, SC-LCE adhesives demonstrated greater performance in probe tack, lap shear, and 180° peel tests at room temperature. Additionally, the switchable adhesion functionality was demonstrated by lifting a 1 kg weight using a 10 × 10 mm *SC3* adhesive film between stainless steel plates, which cleanly debonded upon heating to the isotropic state. This class of reprocessable, switchable LCE adhesives represents a promising platform for applications in reusable consumer products, medical devices, and manufacturing.

## Supplementary Material





## Notes

Synthesis; NMR and FRIR spectra; calculations; DSC thermograms; DMA data; and lap shear data (PDF)
Lap shear demonstration (MOV)
