# Supplementary material for: Switchable Pressure-Sensitive Adhesion in Nematic Side-Chain Liquid Crystal Elastomers
Source: Macromolecules. 2025 Oct 9;58(22):12191–200. doi: 10.1021/acs.macromol.5c01692 (PMC12659420; doi:10.1021/acs.macromol.5c01692)
Supplement: Supplementary file 1 [file ma5c01692_si_001.pdf]

Supporting information

Switchable Pressure-Sensitive Adhesion in Nematic Side-Chain Liquid Crystal Elastomers

Noboru Koshimizu[1,2], *Mohand O. Saed*[1]*

[1] Cavendish Laboratory, University of Cambridge, J.J. Thomson Avenue, Cambridge, CB3 0HE, United Kingdom

[2] Electronic and Imaging Materials Res. Labs, Toray Industries, Inc., 3-2-1 Sonoyama, Otsu, Shiga 530-0842, Japan

* Corresponding author: mos29@cam.ac.uk.

Table of Contents

S1. Synthesis and Characterization 2

S1-1. Synthesis 2

S1-2. ¹H NMR and FTIR Data 3

S2. Phase Transition Behaviors and Adhesive Properties 10

References 13

S1. Synthesis and Characterization

S1-1. Synthesis

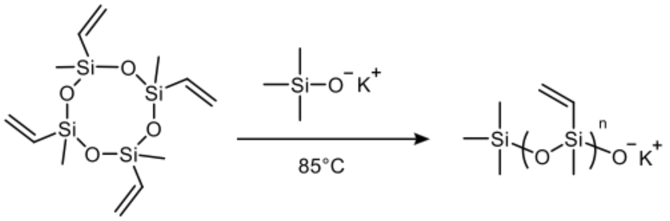

Scheme S1. Synthesis of poly(methylvinylsiloxane)

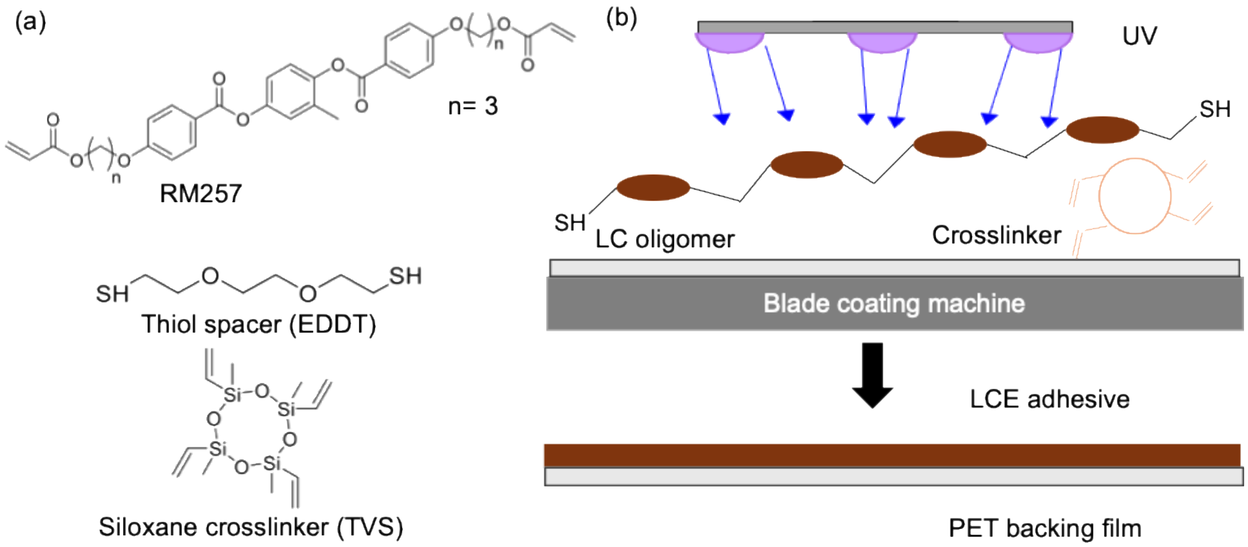

Scheme S2. Synthesis and preparation of main-chain liquid crystal elastomer (MC). The synthesis and preparation including the adhesive tape were carried out following our previously reported method.[1]

S1-2. ^{1}H NMR and FTIR data

4-cyanophenyl 4-(bromomethyl)benzoate:

^{1}H NMR (400 MHz, $CDCl_3$) δ (ppm) = 8.21 (2H, *d*, *J* = 8.0 Hz), 7.78 (2H, *d*, *J* = 8.0 Hz), 7.58 (2H, *d*, *J* = 8.0 Hz), 7.40 (2H, *d*, *J* = 8.0 Hz), 4.69 (2H, *s*).

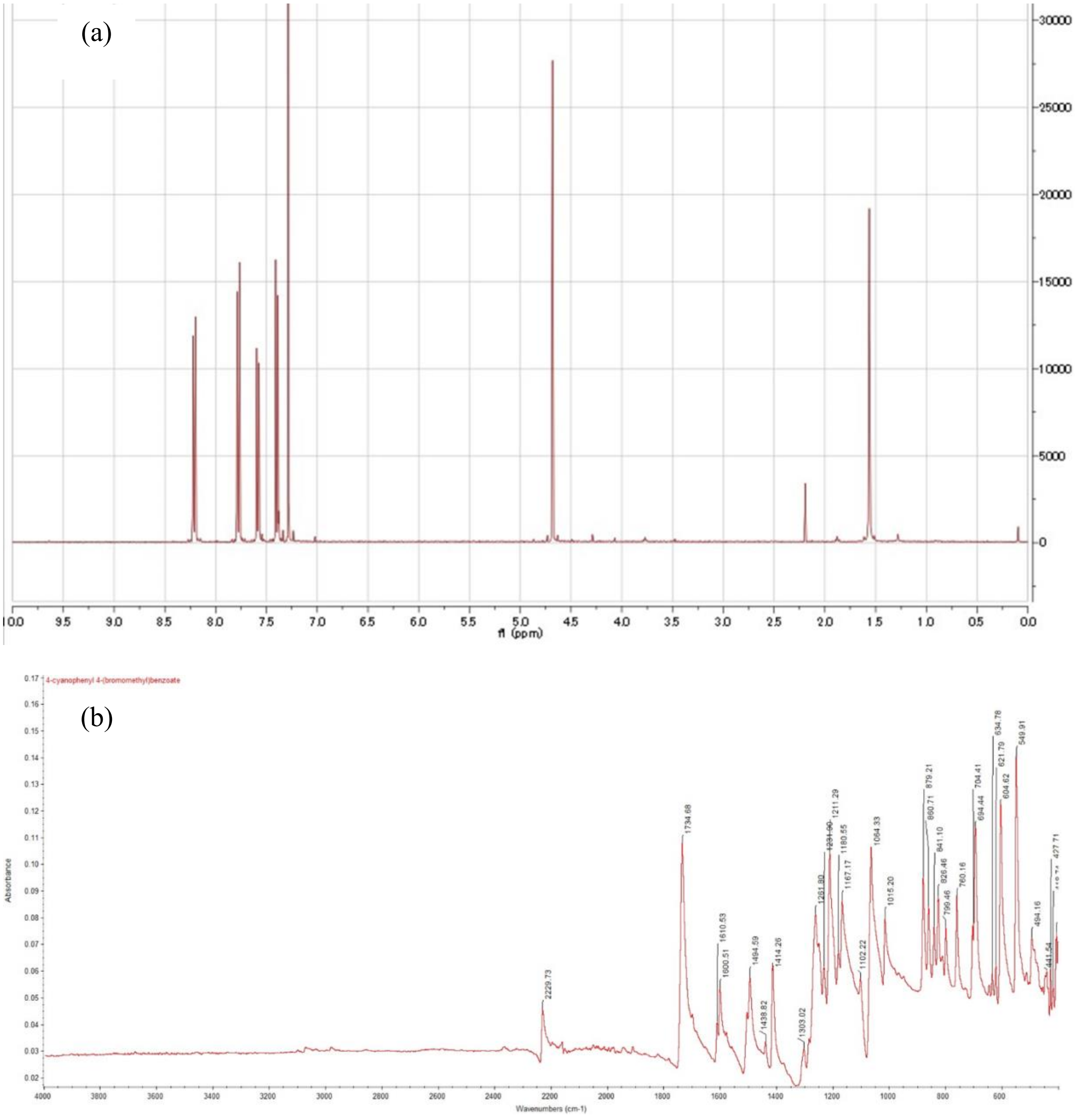

Figure S1. a) ^{1}H NMR and b) FTIR spectrum of 4-cyanophenyl 4-(bromomethyl)benzoate.

2-(4-((4-cyanophenoxy)carbonyl)benzyl)isothiouronium bromide:

^{1}H NMR (400 MHz, DMSO-d) δ (ppm) = 9.23 (2H, *s*), 9.01 (2H, *s*), 8.16 (2H, *d*, *J* = 8.0 Hz), 8.00 (2H, *d*, *J* = 8.0 Hz), 7.66 (2H, *d*, *J* = 8.0 Hz), 7.56 (2H, *d*, *J* = 8.0 Hz), 4.62 (2H, *s*).

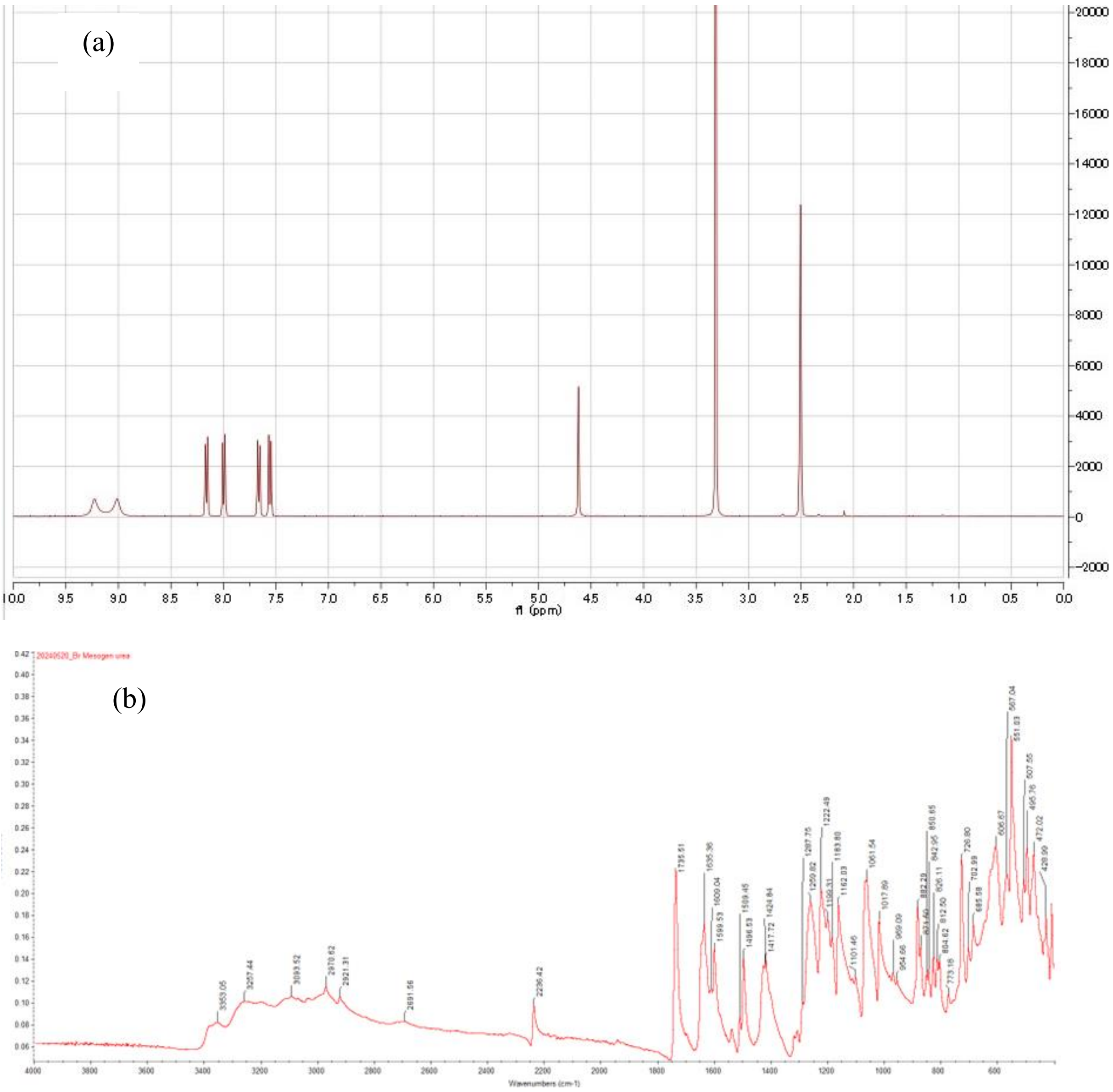

Figure S2. a) ^{1}H NMR and b) FTIR spectrum of 2-(4-((4-cyanophenoxy)carbonyl)benzyl)isothiouronium bromide.

4-cyanophenyl 4-(mercaptomethyl)benzoate:

^{1}H NMR (400 MHz, $CDCl_3$) δ (ppm) = 8.17 (2H, *d*, *J* = 8.0 Hz), 7.77 (2H, *d*, *J* = 8.0 Hz), 7.51 (2H, *d*, *J* = 8.0 Hz), 7.39 (2H, *d*, *J* = 8.0 Hz), 3.85 (2H, *d*, *J* = 8.0 Hz), 1.85 (1H, *t*, *J* = 8.0 Hz).

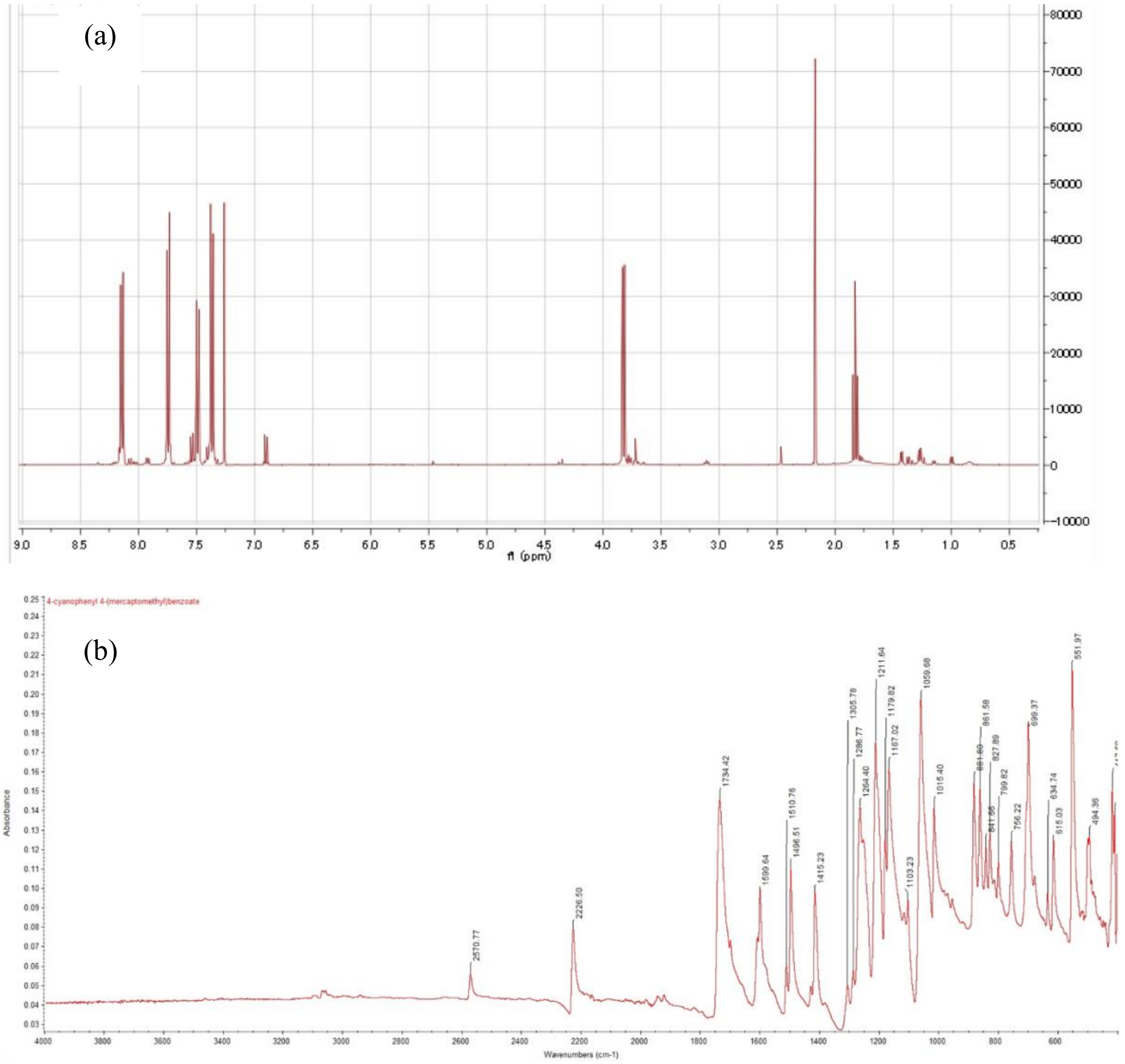

Figure S3. a) ^{1}H NMR and b) FTIR spectrum of 4-cyanophenyl 4-(mercaptomethyl)benzoate.

FTIR characterization of SCn samples:

When the amount of catalyst or photo-initiator (PI) was changed from the values written in Experimental Section, the changed values relative to all components except for toluene are added in parentheses (e.g. SC3 (0.12 w% for catalyst)). FTIR spectrums show that more 21 w% and 19

wt% unreacted thiols remain in SC3 (0.4 w% for PI) and SC3 (0.2 w% for PI) respectively (Thiol peak is at 2571 cm^{-1}) than that of SC3. The insufficient amount of PI is assumed to be the cause. Therefore, 0.5 w% of PI was chosen for this study.

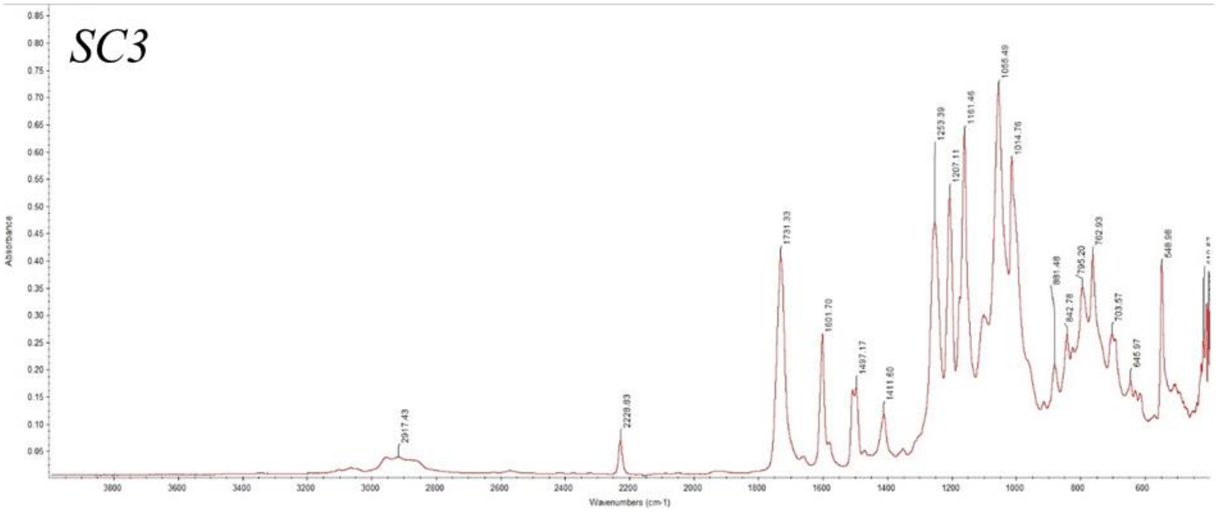

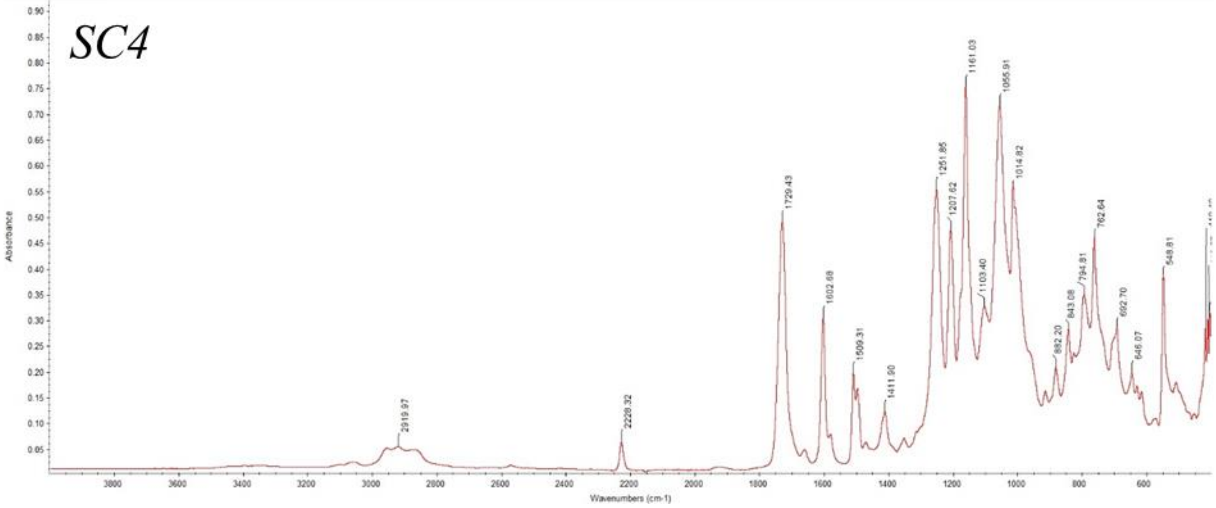

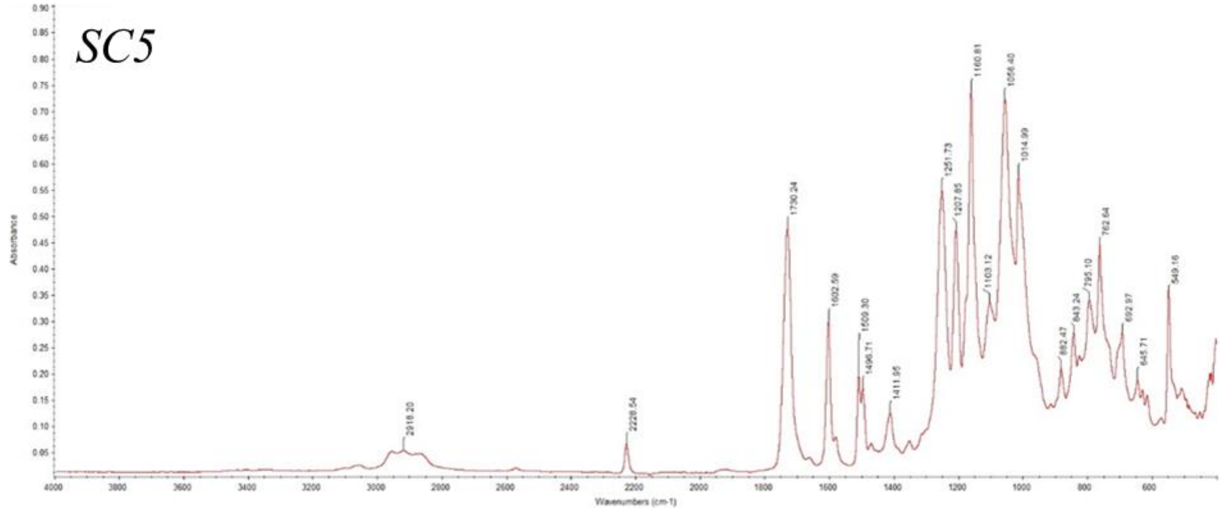
SC5
Absorbance
Wavenumbers (cm-1)

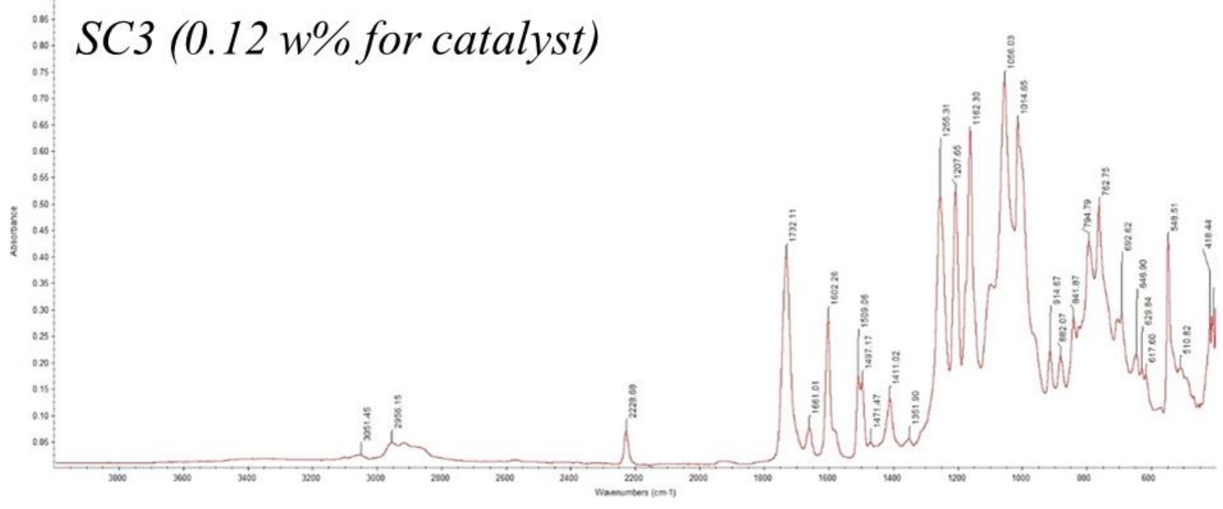
SC3 (0.12 w% for catalyst)
Absorbance
Wavenumbers (cm-1)

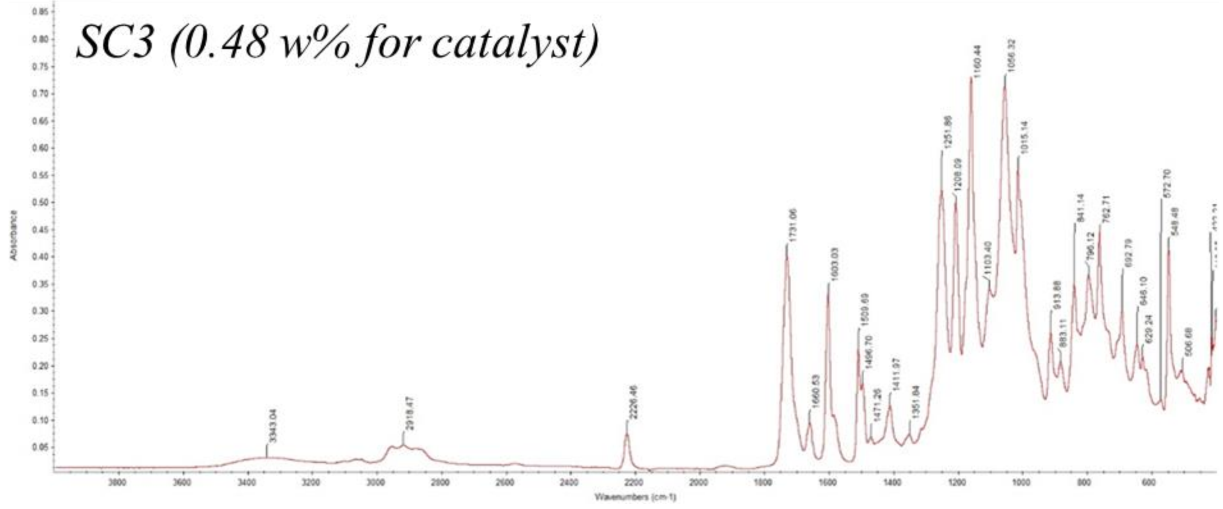
SC3 (0.48 w% for catalyst)
Absorbance
Wavenumbers (cm-1)

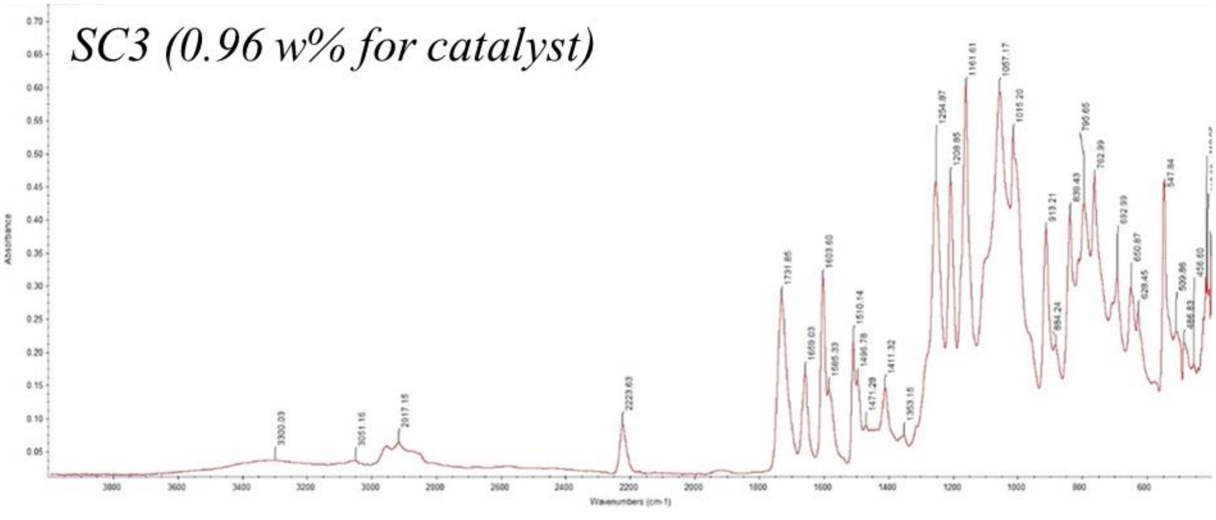
SC3 (0.96 w% for catalyst)
Absorbance
Wavenumbers (cm-1)

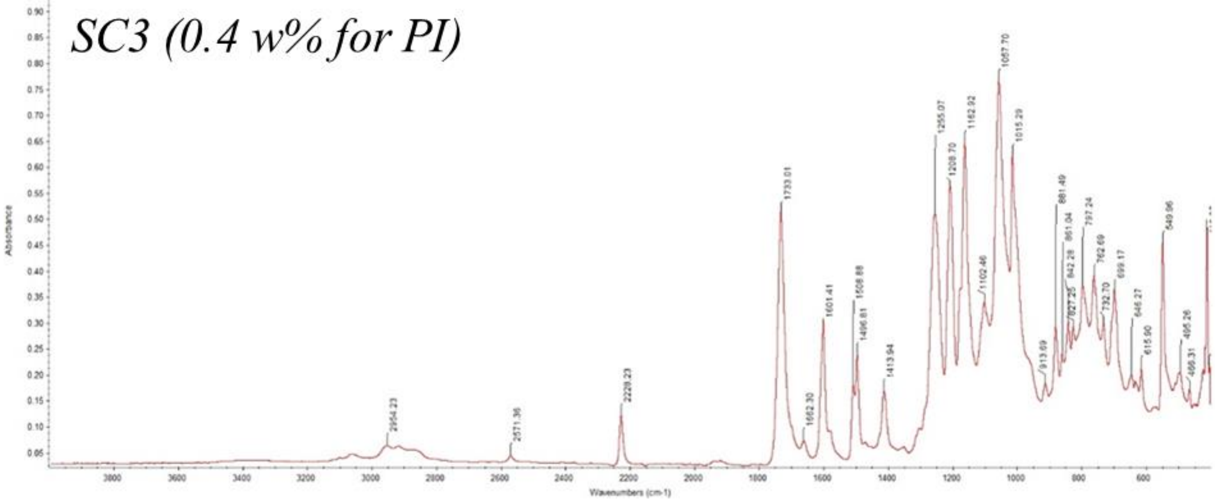
SC3 (0.4 w% for PI)
Absorbance
Wavenumbers (cm-1)

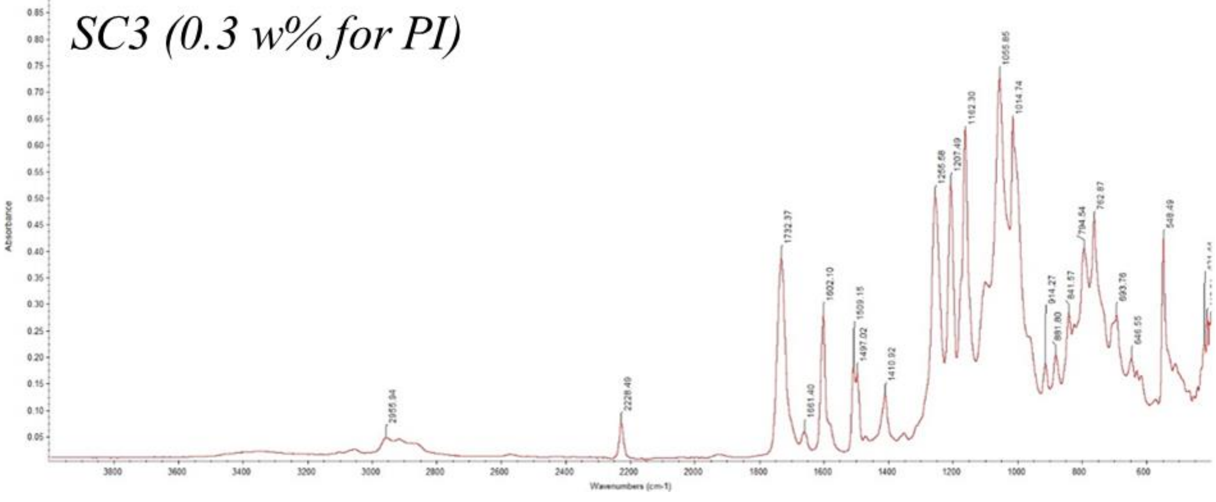
SC3 (0.3 w% for PI)
Absorbance
Wavenumbers (cm-1)

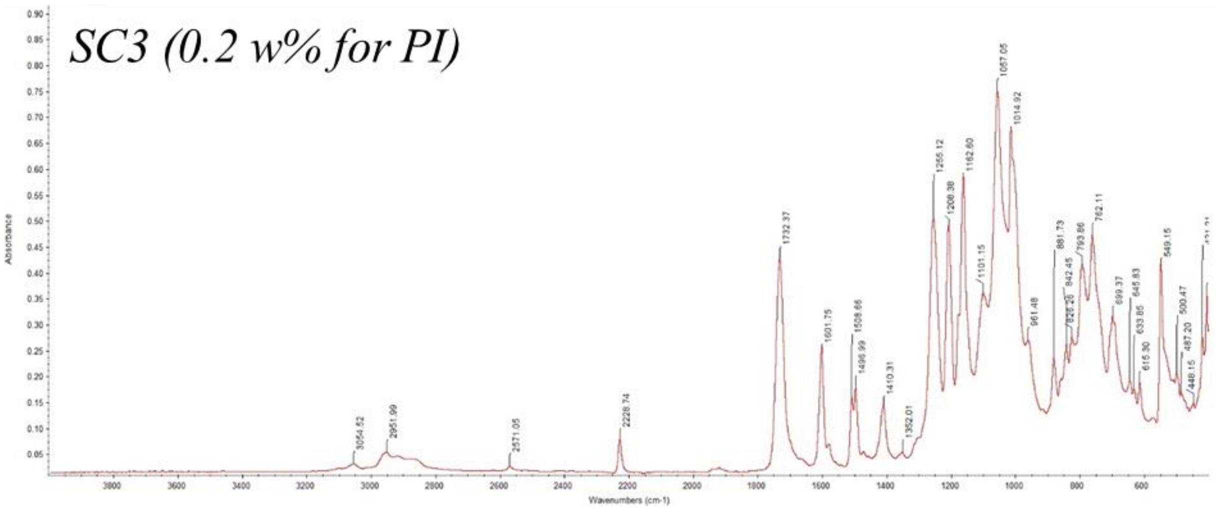

Figure S4. FTIR spectrums of SCn.

S2. Phase Transition Behaviors and Adhesive Properties

Table S1. DMA and DSC results

Sample	The EDDET-to-RM257 molar ratios	DMA		DSC	
		Tg (°C)	tan δ at Tg	Tg (°C)	Tni (°C)
SC3	4-3	6.3	2.81	-14.1	108.2
SC4	5-4	9.6	2.14	-12.3	-
SC5	6-5	14.0	2.13	-14.3	-
SC3 (0.12 w% for catalyst)	4-3	11.0	2.08	-14.8	107.4
SC3 (0.48 w% for catalyst)	4-3	5.8	1.72	-12.5	-
SC3 (0.96 w% for catalyst)	4-3	7.4	1.62	-11.5	106.5
SC3 (0.4 w% for PI)	4-3	8.9	1.86	-8.4	-
SC3 (0.3 w% for PI)	4-3	8.3	1.54	-12.0	-
SC3 (0.2 w% for PI)	4-3	N/D*	N/D*	-20.1	106.1
MC	2-1	2.3	1.07	-9.7	57.4
VHB	-	11.5	1.23	-	-

*It was too soft to be measured.

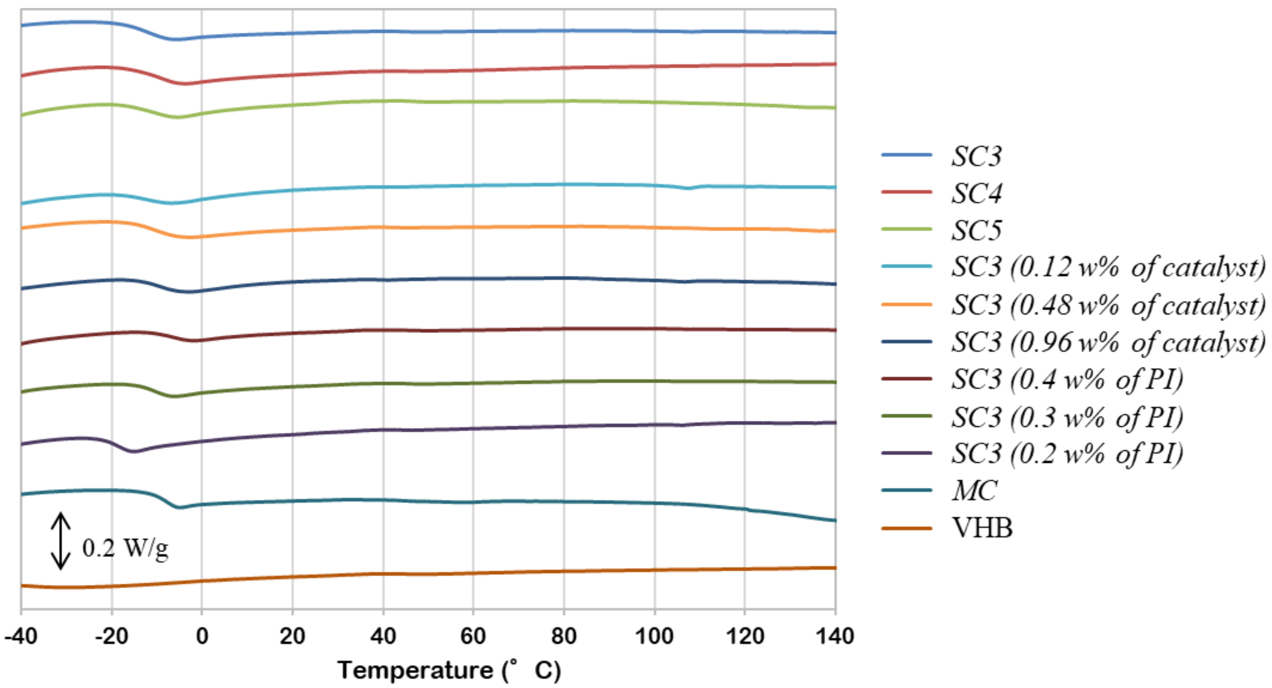

Figure S5. DSC traces of free standing adhesive films. The samples were heated to 150 °C at 10 °C min-1, held isothermally for 5 min, cooled to −50 °C at 10 °C min-1, and held isothermally for 5 min. This cycle was repeated three times and third heating cycle was read for Tg and T_{NI}.

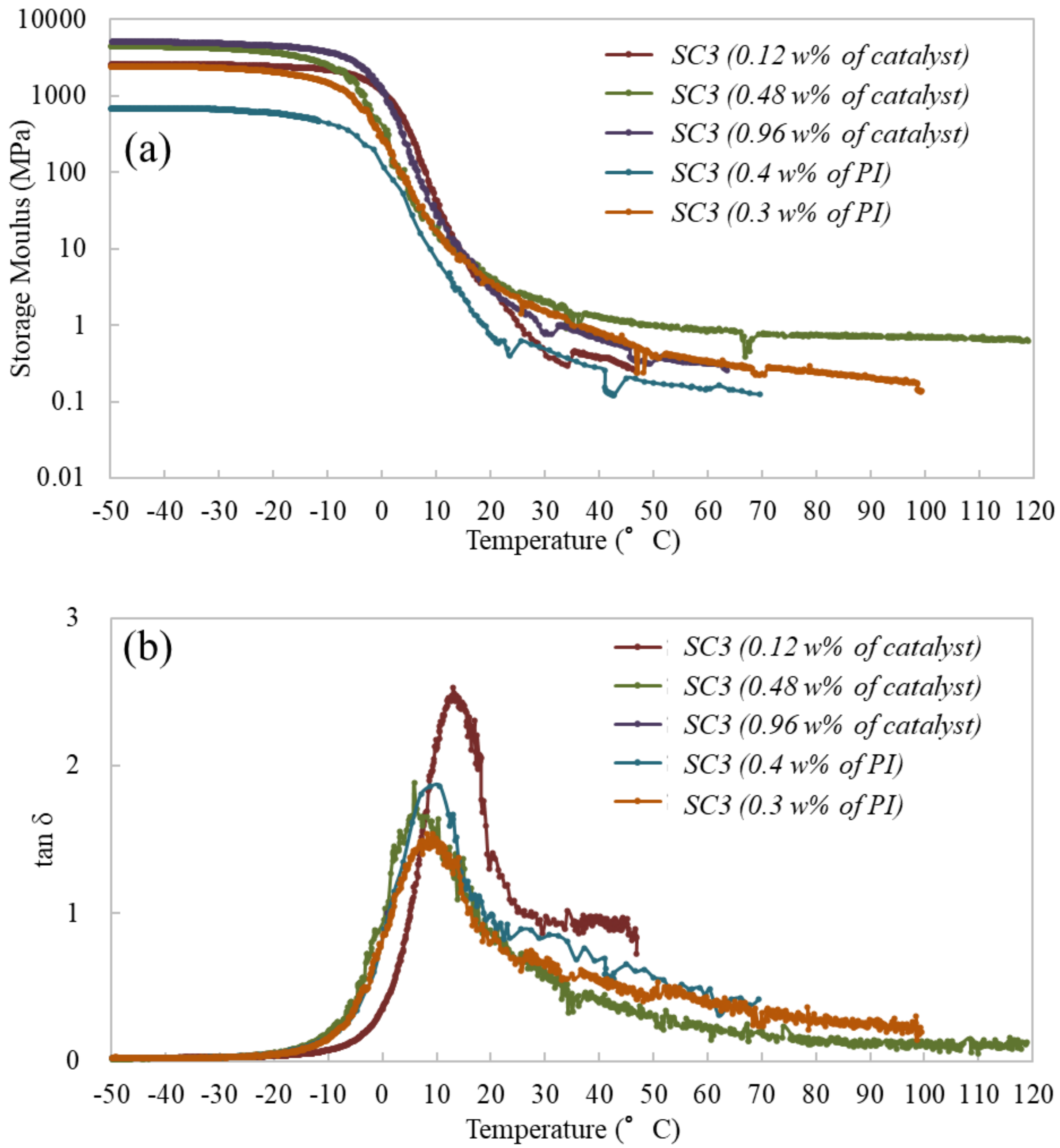

Figure S6. Dynamic soft elasticity: a) The storage modulus G', and b) the loss factor tan δ, at fixed frequency ω = 1.0 Hz, with a strain amplitude of 0.02 %, on sample heating at 2 °C min^{-1}.

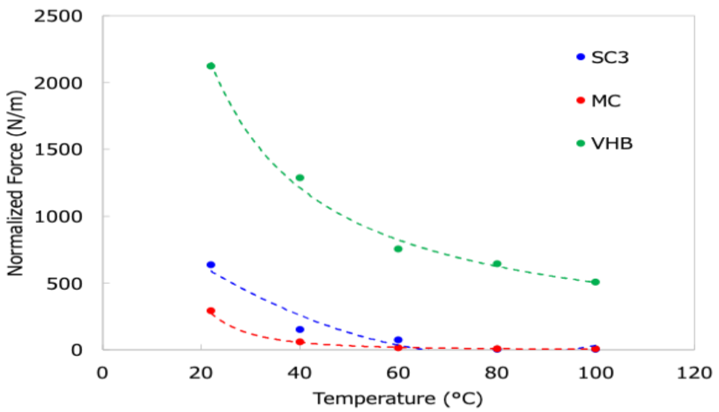

Figure S7. Peel testing as a function of temperature for SC3, MC-LCE, and VHB

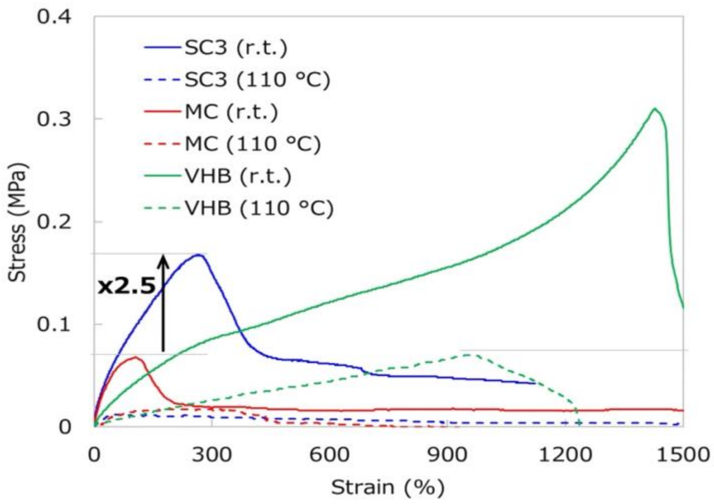

Figure S8. Lap shear testing as a function of temperature. Shear stress vs. shear strain curves for SC3, MC-LCE, and VHB measured at room temperature (nematic phase) and at 110 °C (isotropic phase).

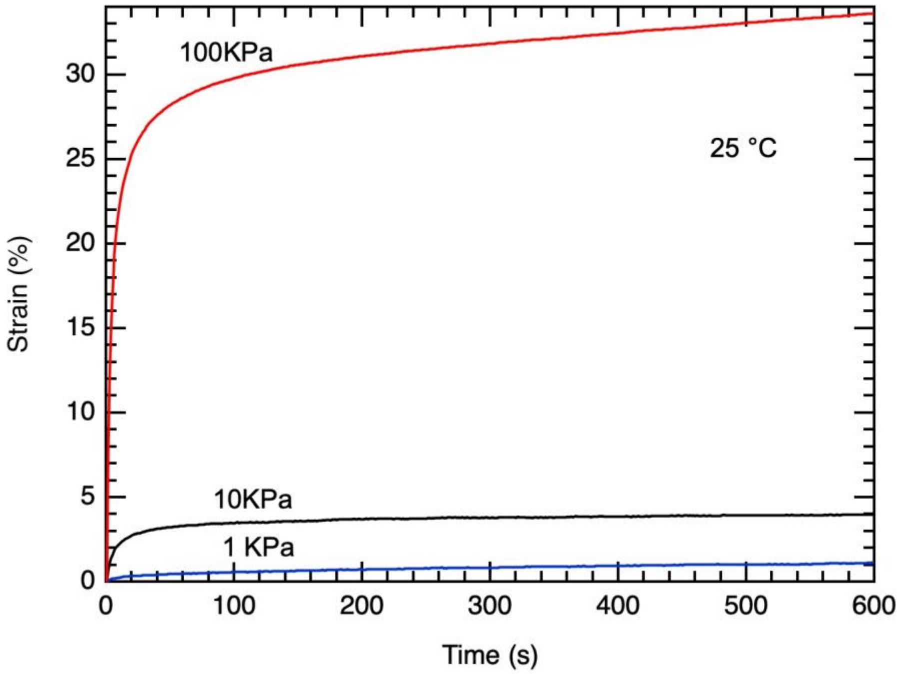

Figure S9. Creep testing of SC3 specimen under various applied stress at toom temperature.

Reference:

(1) Guo, H.; Saed, M. O.; Terentjev, E. M. Mechanism of Pressure-Sensitive Adhesion in Nematic Elastomers. *Macromolecules* **2023**, *56* (16). https://doi.org/10.1021/acs.macromol.3c01038.
